# Associations of Delay in Doctor Consultation With COVID-19 Related Fear, Attention to Information, and Fact-Checking

**DOI:** 10.3389/fpubh.2021.797814

**Published:** 2021-12-13

**Authors:** Agnes Yuen-Kwan Lai, Shirley Man-Man Sit, Socrates Yong-Da Wu, Man-Ping Wang, Bonny Yee-Man Wong, Sai-Yin Ho, Tai-Hing Lam

**Affiliations:** ^1^School of Nursing, The University of Hong Kong, Hong Kong, Hong Kong SAR, China; ^2^School of Public Health, The University of Hong Kong, Hong Kong, Hong Kong SAR, China

**Keywords:** COVID-19, coronavirus, infodemic, infodemiology, delay in doctor consultation, patient delay, public health, information and communication technologies

## Abstract

**Background:** Delaying doctor consultation is harmful. Fear of COVID-19 leads to delays in seeking medical care at a time when pandemic information overflows. However, little is known about the role of COVID-19 related fear, attention to information, and fact-checking in such delay.

**Objective:** Under the Hong Kong Jockey Club SMART Family-Link Project, we examined the associations of delay in doctor consultation amidst the pandemic with sociodemographic characteristics, COVID-19 related fear, attention to information, and fact-checking.

**Methods:** We conducted a population-based online cross-sectional survey in May 2020 on Hong Kong Chinese adults. Respondents reported whether the pandemic caused any delay in doctor consultation (yes/no), level of COVID-19 related fear, attention to information and fact-checking (all on a scale of 0 to 10 and recoded into tertiles of low, moderate, high). Regression analyses were used to examine the associations of delay and fear with sociodemographic characteristics, attention and fact-checking, adjusting for covariates. Data were weighted by sex, age and education level of the population.

**Results:** Of 4,551 respondents (46.5% male, 59.7% aged over 45 years), 10.1% reported delay in doctor consultation. The mean score was 6.4 for fear, 8.0 for attention and 7.4 for fact-checking. Delay was more common in males and increased with age and fear. High vs. low level of fear was associated with delay [adjusted odd ratios (AOR) 2.68, 95% confidence interval (CI) 2.08, 3.47]. Moderate level of fact-checking was negatively associated with delay (AOR 0.72, 95% CI 0.56, 0.92). Females reported greater fear and fear decreased with age. Fear increased with attention to information and decreased with fact-checking. Fear substantially mediated the association of delay with attention (96%) and fact-checking (30%).

**Conclusions:** We have first shown that delay in doctor consultation increased with fear of COVID-19 and decreased with fact-checking amidst the pandemic. Fear also increased with attention to COVID-19 related information and decreased with fact-checking. Understanding these associations can help policymakers develop targeted communication and support to the public to reduce delayed doctor consultations and the associated COVID-19-related or unrelated morbidity and mortality in the community.

## Introduction

Delay in doctor consultation can increase morbidity and mortality risk associated with various health conditions, and reduce the effectiveness of treatments (1, 2). Amidst the COVID-19 pandemic, such delay can result in more severe presentations and related excess deaths (3). Fear of infection in healthcare settings has led to delays in or avoidance of medical care, with negative health consequences (4–6). Understanding the environmental and psychosocial factors associated with such delay is needed to reduce fear and delay.

Meanwhile, the simultaneously ongoing *infodemic*, coined by the World Health Organization as an overabundance of information and rapid spread of misinformation, has also undermined the public's ability to discern the truth, thereby complicating public health responses to the pandemic (7, 8). Not only can misinformation fuel fear and confusion, frequent exposure to COVID-19-related information has been associated with mental distress, including fear, anxiety and depression (9–11). During a pandemic, individuals may not be able to obtain high-quality information, even if they regularly fact-check, and especially when they lack knowledge about science or are influenced by negative emotion or misinformation (12).

We used keywords of “COVID-19,” “coronavirus,” “delay,” “patient delay,” “seek” and “doctor consultation” to search PubMed and Cochrane Library up to 26 July 2021. Four surveys reported patient delays amidst the pandemic due to reasons including fear of infection, inability to get an appointment or access the care location, and being discouraged to access care to curb transmission (4, 13–15). We found no survey reports on the role of both fear of COVID-19 and COVID-19 information-related attitude (attention) and behavior (fact-checking) in delay in doctor consultation.

In Hong Kong, one of the most developed and westernized cities in China with a population of over 7 million, about 1,100 confirmed cases and 4 deaths were reported since the first case on 23 January until 31 May 2020 (around the end of the second wave of outbreak) (16). As of 3 November 2021, a total of 12,034 confirmed cases and 213 deaths were reported (17). This could be attributed to ubiquitous voluntary masking, hand hygiene and government public health measures including contact tracing and social distancing, but no lockdown. People were strongly advised against visiting places with high contact risk, including clinics and hospitals unless they had suspicious COVID-19 symptoms (18). Many hospitals also reduced non-emergency services. The prevalence and determinants of delay in doctor consultation in Hong Kong during the pandemic are unknown.

Under the Hong Kong Jockey Club SMART Family-Link Project, we conducted the Family Amidst COVID-19 (FamCov) survey in May 2020, after the second wave of the pandemic was under control. This paper reports (i) the associations of delay in doctor consultation and fear during COVID-19 with sociodemographic characteristics, (ii) the association of delay with fear and COVID-19 information-related attitude (attention) and behavior (fact-checking), (iii) the mediating effects of fear with information-related attitude and behavior on delay in doctor consultation in Hong Kong adults. We hypothesized that such delay is associated with fear of COVID-19 and COVID-19 information-related attitude and behavior, and that fear is a mediator of information-related attitude and behavior on delay in doctor consultation.

## Methods

### Study Design and Sampling

We conducted this online population-based cross-sectional survey by collecting as large a sample as possible with budget constraint and in a very short period of time (6 days from 26 May to 31 May 2020) when the second wave was under control and before another wave began.

**Figure 1:**
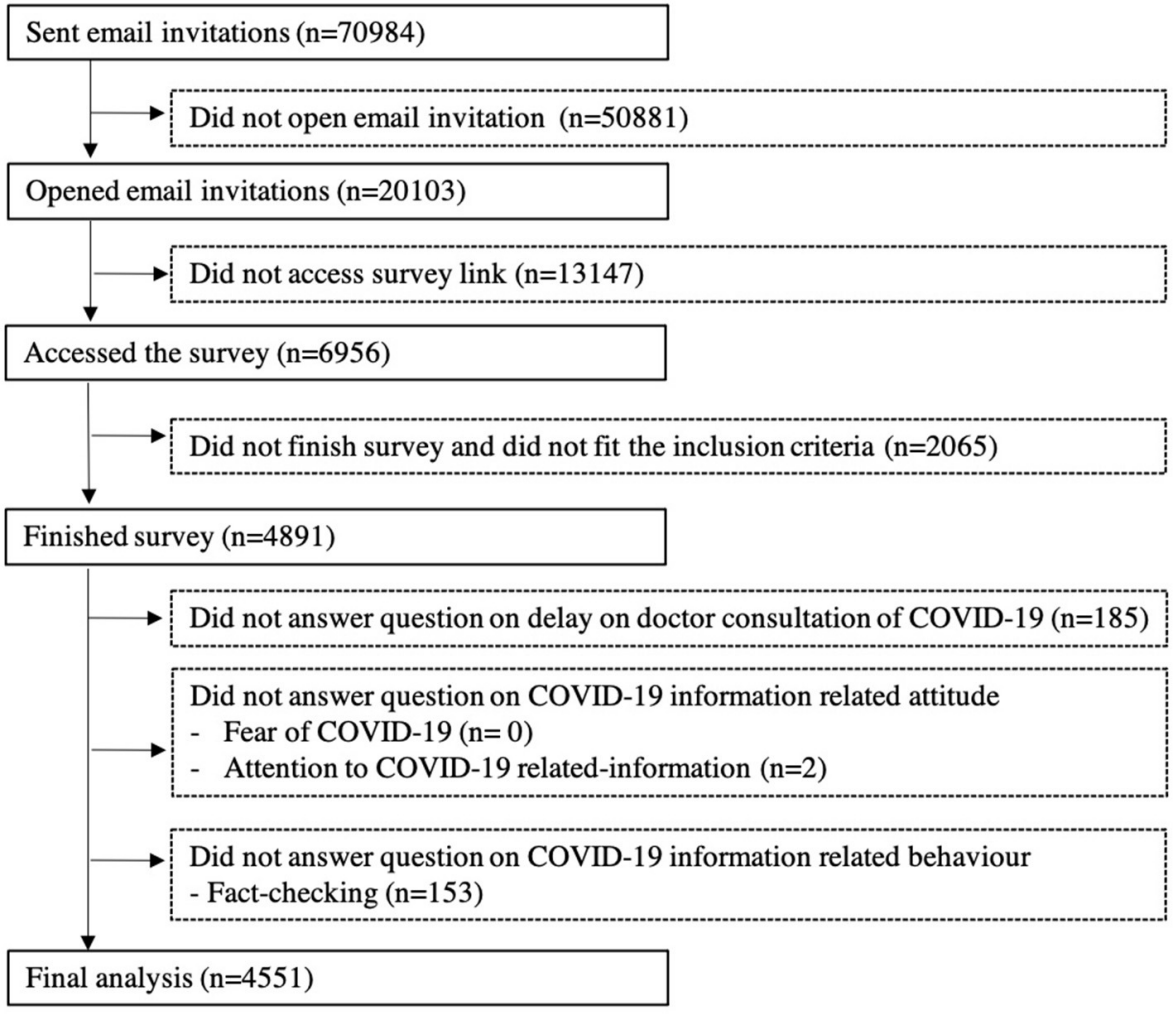
The survey recruitment flow diagram.

Detailed survey design and recruitment procedures have been published in our previous papers (19, 20). Briefly, email invitations were sent to 70,984 adults aged 18 years and above with valid email addresses from both probability and non-probability-based panels by the Hong Kong Public Opinion Research Institute (HKPORI), a well-known local survey agency. Four thousand eight hundred and ninety-one respondents who fit the inclusion criteria completed the survey. The response rate was 24.3% based on 20,103 respondents who opened the email. We excluded 185 respondents who did not answer the question on delay in doctor consultation, 2 respondents who did not answer the question on attention of COVID-19 information, and 153 respondents who did not answer the question on fact-checking of COVID-19 information, respectively. The remaining 4,551 were included in the present analyses. Figure 1 shows the flow diagram. Informed consent was obtained from all respondents before starting the survey. Ethics approval was obtained from the Institutional Review Board of the University of Hong Kong/Hospital Authority Hong Kong West Cluster (IRB reference no.: UW20-238).

### Measurements

Delay in doctor consultation (delay) was assessed as one of 11 answer options to the question, “What harms have COVID-19 brought you?” One or more answer options could be selected. We analyzed “delay in doctor consultation” as “yes” vs. “no.”

Fear is an unpleasant natural emotional response to perceiving or recognizing a threat (such as the pandemic) and serves to keep people away from danger (20). Fear of COVID-19 (fear) was assessed by the question, “Has COVID-19 caused you fear?” on a scale of 0 (no fear at all) to 10 (very fearful), and was used in our previous paper (20). Higher scores indicated greater fear.

Two questions on COVID-19 information-related attitude (attention) and behavior (fact-checking) were asked. Attention is the cognitive process that makes it possible to position oneself toward relevant stimuli and respond to it. Fact-checking is a behavioral process to verify factual information, so as to promote the veracity and correctness of reporting. Attention to COVID-19 information (attention) was assessed by the question, “In general, how much attention do you pay to COVID-19-related information?” on a scale of 0 (none) to 10 (excessive). Higher scores indicated greater attention. Fact-checking of COVID-19 information (fact-checking) was assessed by the question, “When the outbreak was serious, how often did you fact-check COVID-19-related information before forwarding to family members?” on a scale of 0 (never) to 10 (always). Higher scores indicated more fact-checking.

We also collected information on sex, age group (18–24, 25–34, 35–44, 45–54, 55–64, and 65 years or above), education (primary or lower, secondary, diploma or certificate, associate degree, and degree or higher), and household monthly income (no income, less than HKD 4,000, 4,000–9,999, 10,000–19,999, 20,000–29,999, 30,000–39,999, and 40,000 or higher (USD 1 = HKD 7.8).

### Statistical Analysis

Analyses were conducted using Stata version 15.0. Statistical significance was indicated by *P* < 0.05, and marginal statistical significance was indicated by *P* < 0.1. Results on respondent characteristics, presented as mean and standard deviation or number and percentage, were weighted by sex, age group and education of the Hong Kong general population to improve representativeness (21). Several variables were recoded: age (<35, 35–44, 45–54, 55–64 and 65 years or above), education (secondary or below, and post-secondary), household monthly income [lower (less than or equal to the median monthly household income per person) or higher] (19).

For ease of interpretation, we first examined the associations of delay (yes/no) and fear [high (median and above, scores from 7 to 10) / low (below median, scores from 0 to 6)] with sociodemographic characteristics (i.e., sex, age group, education, and household monthly income) by Chi square test, as shown in Table 1. Although only sex and age showed marginally significant associations with delay and fear, education was still considered as a potential confounding factor, as it is one of the key factors influencing health-related decisions and outcomes (22).

**Table 1:** Prevalence of delay in doctor consultation by sociodemographic characteristics.

	**Unweighted**	**Weighted^**a**^**	**Delay in doctor consultation**		**High level of fear of COVID-19** ^**c**^	
	***n* (%)**	**(%)**	***n* (%)**	***P*-value**	***n* (%)**	***P*-value**
**Total**	4,551		454 (10.1)		2,291 (51.1)	
**Sex**				0.07^#^		<0.001^***^
Male	1,966 (43.2)	(46.5)	243 (11.7)		1,004 (48.2)	
Female	2,585 (56.8)	(53.5)	211 (8.8)		1,287 (53.6)	
**Age group, years**				0.052^#^		<0.001^***^
18–34	1,179 (25.9)	(23.3)	88 (8.4)		593 (56.9)	
35–44	1,284 (28.2)	(17)	54 (7.1)		457 (60.0)	
45–54	1,138 (25.0)	(18.3)	109 (13.2)		437 (53.1)	
55–64	756 (16.6)	(19.7)	82 (12.5)		404 (45.5)	
≥65	194 (4.3)	(21.7)	122 (10.1)		401 (41.2)	
**Education**				0.72		0.81
Secondary or below	609 (13.5)	(65.9)	311 (10.6)		1,555 (52.9)	
Post-secondary	3,915 (86.6)	(34.1)	141 (9.3)		723 (47.6)	
**Household monthly income** ^**b**^				0.72		0.46
Lower	1,179 (29.6)	(51.7)	232 (11.6)		1,065 (52.9)	
Higher	2,809 (70.4)	(48.3)	183 (9.7)		949 (50.4)	

a *Data were weighted by sex, age group and education of the 2019 Hong Kong general population*.

b *Income divided by household size dichotomized into ‘lower' (less than or equal to median monthly household income) and “higher”*.

c *Fear of COVID-19 recoded into 2 groups according to its median value, as “Low” (scores from 0 to 6) and “High” (scores from 7 to 10)*.

# *P < 0.1*;

*** *P < 0.001*.

Second, we used logistic regression to examine the associations between delay (yes/no) and levels (high/moderate/low, according to their tertiles) of fear, COVID-19 information related attitude (attention) and behavior (fact-checking) with mutual adjustment and with potential confounding factors, as shown in Table 2. The level of fear was recoded as “Low” (scores from 0 to 5), “Moderate” (6 to 7), and “High” (9 to 10). The level of attention was recoded as “Low” (0 to 7), “Moderate” (8), and “High” (9 to 10). The level of fact-checking was recoded as “Low” (0 to 6), “Moderate” (7 to 8), and “High” (9 to 10).

**Table 2:** The associations of delay in doctor consultation with COVID-19 related fear, attention to information and fact-checking.

	**All**	**Delay in doctor consultation^**a**^**	**AOR^**b**^ (95% CI)**	***P*-value**
	***n* (%)**	***n* (%)**		
**Level of fear of COVID-19 (scores 0–10)**				
Low (0 to 5)	1,724 (38.4)	108 (6.2)	1	
Moderate (6 to 7)	1,238 (27.6)	120 (7.6)	1.31 (0.99, 1.72)	0.06^#^
High (8 to 10)	1,527 (34.0)	241 (16.6)	2.68 (2.08, 3.47)	<0.001^***^
*P* for trend				<0.001^***^
**Level of attention to COVID-19 information (scores 0–10)**				
Low (0 to 7)	1,524 (34.0)	122 (9.2)	1	
Moderate (8)	1,329 (29.6)	149 (9.4)	1.28 (0.98, 1.67)	0.07^#^
High (9 to 10)	1,625 (36.4)	469 (10.1)	1.09 (0.83, 1.42)	0.55
*P* for trend				0.69
**Level of fact-checking of COVID-19 information (scores 0–10)**				
Low (0 to 6)	1,318 (29.4)	129 (11.8)	1	
Moderate (7 to 8)	1,718 (38.3)	174 (10.9)	0.72 (0.56, 0.92)	0.009^**^
High (9 to 10)	1,452 (32.4)	166 (7.7)	0.78 (0.60, 1.02)	0.06^#^
*P* for trend				0.098

a *Data were weighted by sex, age group and education of the 2019 Hong Kong general population*.

b *Mutually adjusted and adjusted for sex, age and education*.

*AOR, adjusted odd ratio; CI, confidence interval*.

# *P < 0.1*;

** *P < 0.01*;

*** *P < 0.001*.

We then used logistic regression to further examine the associations of high fear (vs. moderate/low) with levels (high/moderate/low) of attention and fact-checking, adjusting for each other and potential confounders, as shown in Table 3.

**Table 3:** The associations of level of fear of COVID-19 with attention to information and fact-checking.

	**High level of fear^**a**^ *n* (%)**	**AOR^**b**^ (95% CI)**	***P*-value**
**Level of attention to COVID-19 information (scores 0–10)**			
Low (0 to 7)	542 (35.6)	1	
Moderate (8)	728 (54.8)	2.48 (2.12, 2.91)	<0.001^***^
High (9 to 10)	1,022 (62.5)	4.59 (3.91, 5.38)	<0.001^***^
*P* for trend			<0.001^***^
**Level of fact-checking of COVID-19 information (scores 0–10)**			
Low (0 to 6)	671 (46.1)	1	
Moderate (7 to 8)	925 (53.9)	0.93 (0.79, 1.09)	0.320
High (9 to 10)	759 (52.3)	0.66 (0.56, 0.78)	<0.001^***^
*P* for trend			<0.001^***^

a *Data were weighted by sex, age group and education of the 2019 Hong Kong general population*.

b *Mutually adjusted and adjusted for sex, age, and education*.

*AOR, adjusted odd ratio; CI, confidence interval*.

*** *P < 0.001*.

Lastly, the associations of attention and fact-checking with delay were examined by Analysis of Moment Structures (AMOS; software version 25.0) using the asymptotically distribution free (ADF) method with no normal distribution assumption (23). The mediating effects of fear on attention and fact-checking on delay were performed by Sobel test (24), as shown in Table 4.

**Table 4:** The mediating effect of fear for the associations of attention and fact-checking with delay in consultation.

	**Total effect**	**Direct effect**	**Indirect effect**	**Proportion mediated^**a**^**	**Sobel test statistics**	***P*-value**
Attention to COVID-19 information	0.051	0.002	0.049	96%	8.703	<0.001^***^
Fact-checking of COVID-19 information	−0.027	−0.019	−0.008	30%	−3.330	<0.001^***^

a *Proportion mediated = (indirect effect/total effect) × 100%*.

*** *P < 0.001*.

## Results

### Characteristics of the Survey Sample

Figure 1 shows the recruitment flow. Table 1 shows that of the 4,551 respondents, after weighting, 46.5% were male, 59.7% were aged 45 years or above, 34.1% had attained post-secondary education, and 48.3% had higher household income. 10.1% of respondents reported delay as a perceived harm amidst COVID-19. The mean ± SD score of fear was 6.4 ± 2.3. Of the two COVID-19 information questions, attention scored 8.0 ± 1.5 and fact-checking scored 7.4 ± 2.1.

### The Associations of Delay in Doctor Consultation and Fear With Sociodemographic Characteristics

Table 1 shows that delay was more common in males (*P* = 0.07) and increased with age (*P* for trend = 0.052). No associations of delay with education and household income were reported. Females reported greater fear (*P* < 0.001) and fear decreased with age (*P* for trend < 0.001). No associations of delay and fear with education and household monthly income were reported.

### The Associations of Delay in Doctor Consultation With Fear and Information-Related Attitude and Behavior

Table 2 shows that delay was reported by 6.2, 7.6, and 16.6% respondents with low, moderate and high levels of fear; 9.2, 9.4, and 10.1% respondents with low, moderate and high levels of attention; and 11.8, 10.9, and 7.7% respondents with low, moderate and high levels of fact-checking, respectively.

Delay increased with fear (*P* for trend < 0.001). More respondents with a moderate or high level of fear reported delay than those with a low level of fear [adjusted odd ratios (AOR), 95% confidence interval (CI): 1.31 (0.99, 1.72), *P*= 0.06 and AOR (95 CI): 2.68 (2.08, 3.47), *P* < 0.001, respectively]. More respondents with a moderate level of attention reported delay than those with a low level of attention [AOR (95% CI): 1.28 (0.98, 1.67), *P* = 0.07]. Delay decreased with fact-checking (*P* for trend < 0.1). Fewer respondents with a moderate or high level of fact-checking reported delay than those with a low level of fact-checking [AOR (95% CI): 0.72 (0.56, 0.92), *P* < 0.01 and AOR (95% CI): 0.78 (0.60, 1.02), *P* = 0.06, respectively].

### The Associations of Level of Fear of COVID-19 With COVID-19 Information-Related Attitude and Behavior

Table 3 shows a high level of fear was reported in 35.6, 54.8, and 62.5% respondents with low, moderate and high levels of attention; and 49.4, 53.7, and 52.9% respondents with low, moderate and high levels of fact-checking, respectively. Fear increased with attention (*P* for trend < 0.001) and decreased with fact-checking (*P* for trend < 0.001). More respondents with a moderate or high level of attention reported a high level of fear than those with a low level of attention [AOR (95% CI): 2.48 (2.12, 2.91), *P* < 0.001 and 4.59 (3.91, 5.38), *P* < 0.001, respectively]. Fewer respondents with a high level of fact-checking reported a high level of fear than those with a low level of fact-checking [AOR (95% CI): 0.66 (0.56, 0.78), *P* < 0.001].

### The Mediating Effects of Fear With Information-Related Attitude and Behavior on Delay in Doctor Consultation

Table 4 shows that fear substantially mediated associations of attention (96%) (Sobel test 8.73, *P* < 0.001) and fact-checking (30%) (Sobel test −3.33, *P* < 0.001) on delay.

## Discussion

Our study is the first to show associations of delay in doctor consultation with fear of COVID-19 and COVID-19 information-related attitude (attention) and behavior (fact-checking) amidst the pandemic. Our findings show that one-tenth of respondents experienced delays in doctor consultation. Delay was more common in males and increased with age and fear. Fear was more common in females, decreased with age and fact-checking and increased with attention.

One-tenth of respondents also reported delay in doctor consultation as a perceived harm, which aligns with reports of decreased hospital admissions and visits for a wide spectrum of health conditions since the start of the pandemic in Hong Kong (25, 26) and elsewhere (27, 28). Respondents in Hong Kong reported delays in doctor consultation, which was relatively lower than the estimated 41% of U.S. adults who delayed or avoided medical care (13). This could be explained by the COVID-19 outbreaks that have been better controlled in Hong Kong compared with the U.S. (29).

Many studies report men are more likely to delay, or altogether avoid, doctor consultations, which is associated with the culture of masculinity and social structure of gender and power, including being conditioned to not show signs of weakness or dependence, with health issues (30, 31). Men are less likely to engage in behaviors that promote health and longevity (32), and traditional masculine ideals and a sense of self-worth tied to perceived masculinity are associated with healthcare avoidance and poorer health outcomes (30). Our results showed females and younger age groups had greater fear of COVID-19, consistent with pandemic-related reports including our previous paper (33–36).

Delay increasing with age could be explained by older people likely having more medical issues and appointments, and requiring assistance to seek care (37), which may result in a greater chance for delay. Older people may need more assistance (such as transportation and/or escorting) to attend medical appointments (37). As we hypothesized, greater fear was associated with delay, which is consistent with previous reports (4, 5, 13, 27).

Our results showed delay associated with moderate attention to COVID-19 information. This is consistent with other reports that show exposure and attention to COVID-19 information are positively associated with risk perception (38), including perceived susceptibility and severity, and mental distress (9–11, 39), which may result in delay. However, our findings showed no association of delay with too much attention, which could be due to differences in help-seeking behaviors for some individuals who independently search the internet for health-related information (40).

Our results also highlight the importance of fact-checking any received information related to the pandemic against trustworthy sources. Both the United Nations (UN) and World Health Organization (WHO) have led global efforts to combat misinformation (41, 42). In particular, the WHO has launched the joint “Stop the Spread” global campaign with the United Kingdom government to raise awareness about the risk of misinformation and encourage fact-checking with trusted sources such as national health authorities (43). In Hong Kong, distrust and criticism toward the government facilitated the rapid spread of false information and conspiracy theories (44, 45). With concerns that misinformation is spreading faster than the virus itself (46), fact-checking can help assure that people are using the correct information to make informed decisions (47).

There is also a need for simple, easily understandable, and evidence-based health education for the public to fight against misinformation and misunderstandings (48). As the world battles both a pandemic and concurrent infodemic, accurate and reliable information about the virus and related health information is of paramount importance. Authoritative and credible organizations need to lead and be more effective in the fight against the infodemic. Additionally, our results showed fear increased with attention and decreased with fact-checking, which is consistent with previous reports, and also mediated the associations of attention and fact-checking on delay (38, 49). Health messages need to be communicated in a clear and straightforward manner that does not exacerbate anxieties and irrational fears, and helps facilitate health-related decision-making.

The capacity burden on healthcare systems in various countries due to surging outbreaks have prompted governments to push for people to stay home, even when feeling unwell, which has exacerbated a crisis of increased preventable deaths (50, 51). While outbreaks in Hong Kong were relatively under control, with people voluntarily adhering to masking and social distancing guidelines, they were advised against visiting locations with high density and close contact risk (18). Seeking medical attention promptly amidst the pandemic can help with earlier identification of potential cases and prevent further transmission. There is a need for clear and consistent guidelines across all government and health agencies to warn against delay in doctor consultation and seeking medical care. Alternatively, telemedicine could offer a feasible solution to patients unwilling or unable to seek help in person (52).

More understanding on why people choose to delay care and how information-related attitude and behaviors can affect their decision-making is needed. Future studies should examine the underlying reasons for delay in doctor consultation to prevent further exacerbation of outbreaks and increased COVID-19-related or unrelated morbidity and mortality in the community.

## Limitations

Our study had a few limitations. First, the short sampling time frame and online survey method had led to under sampling of older respondents and those without access to the internet. Online surveys commonly suffer from this methodological limitation. Online surveys are completed only by persons who are literate and have access to the internet, and by those who are sufficiently biased to be interested in the topic (53). However, this problem should not be substantial as the key variables showed only small differences between weighted and unweighted results. Second, it could be queried whether the use of a single-item can fully reflect the connotation of complex constructs. However, studies show that single-item measures have acceptable level reliability and reliability, and play its appropriate role in psychology and social science research (54, 55). Although multi-item measures are more preferred by researchers, single-item measures also have its legitimacy in academic research (54). Third, the cross-sectional design of this survey could only show associations and the current findings were based on our hypothesized pathways. Fourth, the self-reported data might be subject to recall and response biases. Fifth, the survey did not assess the reasons for delay and whether the delay actually occurred, as the question was framed as whether respondents viewed delay as a perceived harm of the pandemic, which should be more concerning than delay with no harm. However, the reported prevalence of those that answered yes is consistent with increased delays reported in recent reports (25, 26). Lastly, respondents' brief history of medical illness and COVID-19 related experience (e.g., having friends or family diagnosed with COVID-19) were not collected, which may influence their health-related attitude and behaviors.

## Conclusions

We have first shown that delay in doctor consultation increased with fear of COVID-19 and decreased with fact-checking amidst the pandemic. Fear also increased with attention to COVID-19 related information and decreased with fact-checking. Understanding these associations can help policymakers develop targeted communication and support to the public to reduce delayed doctor consultations and the associated COVID-19-related or unrelated morbidity and mortality in the community.

## Data Availability Statement

The dataset presented in this article is not readily available because the sharing of data to third parties was not mentioned in subjects' consent. Requests to access the dataset should be directed to the corresponding author.

## Ethics Statement

The studies involving human participants were reviewed and approved by Institutional Review Board of the University of Hong Kong/Hospital Authority Hong Kong West Cluster. The patients/participants provided their written informed consent to participate in this study.

## Author Contributions

AL and SS analyzed the data and wrote the first draft. AL, SS, SW, BW, M-PW, S-YH, and T-HL contributed to the conception and design of the study. AL, SS, SW, S-YH, and T-HL interpreted the results. All authors critically revised and approved the final manuscript.

## Funding

The research was funded by the Hong Kong Jockey Club Charities Trust.

## Conflict of Interest

The authors declare that the research was conducted in the absence of any commercial or financial relationships that could be construed as a potential conflict of interest.

## Publisher's Note

All claims expressed in this article are solely those of the authors and do not necessarily represent those of their affiliated organizations, or those of the publisher, the editors and the reviewers. Any product that may be evaluated in this article, or claim that may be made by its manufacturer, is not guaranteed or endorsed by the publisher.

## Abbreviations

COVID-19: the coronavirus disease 2019
FamCov: Family Amidst COVID-19 survey
CI: confidence interval
SD: standard deviation.
