# Corrigendum: Associations of Delay in Doctor Consultation With COVID-19 Related Fear, Attention to Information, and Fact-Checking

**DOI:** 10.3389/fpubh.2022.847603

**Published:** 2022-02-10

**Authors:** Agnes Yuen-Kwan Lai, Shirley Man-Man Sit, Socrates Yong-Da Wu, Man-Ping Wang, Bonny Yee-Man Wong, Sai-Yin Ho, Tai-Hing Lam

**Affiliations:** ^1^School of Nursing, The University of Hong Kong, Hong Kong, Hong Kong SAR, China; ^2^School of Public Health, The University of Hong Kong, Hong Kong, Hong Kong SAR, China

**Keywords:** COVID-19, coronavirus, infodemic, infodemiology, delay in doctor consultation, patient delay, public health, information and communication technologies

In the original article, there was an error. **The incorrect adjusted odds ratio was included in the abstract for the association between fact-checking and delay**.

A correction has been made to ***Abstract***, ***Results***, ***1***:

The incorrect text stated: Results: Of 4,551 respondents (46.5% male, 59.7% aged over 45 years), 10.1% reported delay in doctor consultation. The mean score was 6.4 for fear, 8.0 for attention and 7.4 for fact-checking. Delay was more common in males and increased with age and fear. High vs. low level of fear was associated with delay [adjusted odd ratios (AOR) 2.68, 95% confidence interval (CI) 2.08, 3.47]. Moderate level of fact-checking was negatively associated with delay (AOR 1.28, 95% CI 0.98, 1.67). Females reported greater fear and fear decreased with age. Fear increased with attention to information and decreased with fact-checking. Fear substantially mediated the association of delay with attention (96%) and fact-checking (30%).

The corrected text appears below:

Of 4,551 respondents (46.5% male, 59.7% aged over 45 years), 10.1% reported delay in doctor consultation. The mean score was 6.4 for fear, 8.0 for attention and 7.4 for fact-checking. Delay was more common in males and increased with age and fear. High vs. low level of fear was associated with delay [adjusted odd ratios (AOR) 2.68, 95% confidence interval (CI) 2.08, 3.47]. Moderate level of fact-checking was negatively associated with delay (AOR 0.72, 95% CI 0.56, 0.92). Females reported greater fear and fear decreased with age. Fear increased with attention to information and decreased with fact-checking. Fear substantially mediated the association of delay with attention (96%) and fact-checking (30%).

The authors apologize for this error and state that this does not change the scientific conclusions of the article in any way. The original article has been updated.

## Publisher's Note

All claims expressed in this article are solely those of the authors and do not necessarily represent those of their affiliated organizations, or those of the publisher, the editors and the reviewers. Any product that may be evaluated in this article, or claim that may be made by its manufacturer, is not guaranteed or endorsed by the publisher.

